# URB2 as an important marker for glioma prognosis and immunotherapy

**DOI:** 10.3389/fphar.2023.1113182

**Published:** 2023-03-24

**Authors:** Chaoyou Fang, Zeyu Zhang, Yongquan Han, Houshi Xu, Zhengyang Zhu, Yichao Du, Pinpin Hou, Ling Yuan, Anwen Shao, Anke Zhang, Meiqing Lou

**Affiliations:** ^1^ Department of Neurosurgery, Shanghai General Hospital, Shanghai Jiao Tong University School of Medicine, Shanghai, China; ^2^ Department of Neurosurgery, Renji Hospital, School of Medicine, Shanghai Jiao Tong University, Shanghai, China; ^3^ Department of Neurosurgery, The Affiliated Hospital of Guizhou Medical University, Guiyang, China; ^4^ Central Laboratory, Renji Hospital, School of Medicine, Shanghai Jiao Tong University, Shanghai, China; ^5^ Department of Neurosurgery, Second Affiliated Hospital, School of Medicine, Zhejiang University, Zhejiang, China; ^6^ Clinical Research Center for Neurological Diseases of Zhejiang Province, Hangzhou, China

**Keywords:** URB2, glioma, immunity, prognosis, immunotherapy

## Abstract

**Introduction:** Glioma is the most common primary brain tumor and primary malignant tumor of the brain in clinical practice. Conventional treatment has not significantly altered the prognosis of patients with glioma. As research into immunotherapy continues, glioma immunotherapy has shown great potential.

**Methods:** The clinical data were acquired from the Chinese Glioma Genome Atlas (CGGA) database and validated by the Gene Expression Omnibus (GEO) database, The Cancer Genome Atlas (TCGA) dataset, Clinical Proteomic Tumor Analysis Consortium (CPTAP) database, and Western blot (WB) analysis. By Cox regression analyses, we examined the association between different variables and overall survival (OS) and its potential as an independent prognostic factor. By constructing a nomogram that incorporates both clinicopathological variables and the expression of URB2, we provide a model for the prediction of prognosis. Moreover, we explored the relationship between immunity and URB2 and elucidated its underlying mechanism of action.

**Results:** Our study shows that URB2 likely plays an oncogenic role in glioma and confirms that URB2 is a prognostic independent risk factor for glioma. Furthermore, we revealed a close relationship between immunity and URB2, which suggests a new approach for the immunotherapy of glioma.

**Conclusion:** URB2 can be used for prognosis prediction and immunotherapy of glioma.

## 1 Introduction

Glioma accounts for approximately thirty percent of brain tumors and eighty percent of malignant brain tumors and is the most frequent primary brain tumor (Omuro and DeAngelis, 2013; Ostrom et al., 2015). According to the criteria of the World Health Organization (WHO), glioma is classified into four different groups, which are associated with malignancy (Ostrom et al., 2017; Wesseling and Capper, 2018). Although aggressive therapies, including debulking surgery, chemotherapy, and external beam radiation therapy, are available, glioma patients currently face a dismal prognosis (Stewart, 2002). Furthermore, systemic medications do not reach therapeutic concentrations inside solid tumors and cause systemic side effects (Blakeley, 2008; Sriraman et al., 2014). Hence, further research on the potential mechanisms of gliomas is imperative.

In recent years, glioma patients have increasingly chosen targeted therapy as a treatment option. Previous studies have revealed a high degree of immune infiltration in glioma (Bush et al., 2017). Numerous mechanisms are involved in the highly inhibited immune function in the glioma microenvironment, including immune checkpoint inhibitors (ICIs) (Ghouzlani et al., 2021). Immune checkpoints (ICs) are costimulators or cosuppressors required to produce an immune response (Korman et al., 2006). There is no doubt that the discovery of immune checkpoints such as CTLA-4 and PD-1 has exerted a significant boost in cancer immunotherapy development and has emerged as a potential treatment option for glioma (Ghouzlani et al., 2021). A breakthrough in glioma treatment by affecting immune checkpoints is being made.

In yeast, URB2 (URB2 ribosome biogenesis homolog) localizes to the nucleolus and encodes a protein measuring 135.2 kDa, which is essential for ribosome biogenesis. As it is critical for the biogenesis of the 60 S subunit, a mutation or depletion of URB2 will disrupt ribosomal subunits and rRNAs (Rosado et al., 2007). However, to date, no study has addressed the specific roles of URB2 in tumorigenesis and progression. Therefore, we investigated the predictive value of URB2 in glioma and elucidated its relationship with immunity in this study. Moreover, GSEA was conducted to confirm URB2-related biological functions and signaling pathways. To better understand the immunological correlates of URB2, we evaluated the relationship between URB2 expression and prognosis related to immune infiltration and the tumor microenvironment. This study is expected to lead to the development of novel therapies and provide effective clinical biomarkers for glioma.

## 2 Materials and methods

### 2.1 Cell culture

The U87 and U251 human malignant glioblastoma cell lines were purchased from the China Infrastructure of Cell Line Resources (Beijing, China). Cells were cultured in complete DMEM/F12 medium (2.5% certified fetal bovine serum, FBS (Vivacell, Shanghai, China), 15% horse serum, and a 1% antibiotic mixture) under 5% CO_2_ and 37°C. The medium was changed every 3–4 days, and cultures were split using 0.25% trypsin. All experiments were carried out on cells with viability >95%. The cell lines were authenticated at VivaCell Shanghai using short tandem repeat analysis.

### 2.2 Transfection of siRNA

U87 and U251 cells in 6-well plates (about 5 × 10^5^ cells/well) were transfected with siURB2 or corresponding negative controls. Lipo3000 transfection reagent was simultaneously added into the medium for efficient transfection. After 6 h, we replaced the culture medium. Detection was made 24 h after transfection. The human targeting siRNA of URB2 was purchased from sigama-aldrich.

### 2.3 Cell viability assay

The viability of glioma cells was evaluated using Cell Counting Kit-8 (CCK-8; cat. No. CK04; Dojindo Molecular Technologies, Inc.). U87 and U251 cells were seeded in 96-well plates (100 µl containing 3,000 cells/well). Cells were cultured in DMEM at 37°C under 5% CO_2_ conditions for 24, 48 or 72 h. CCK-8 solution (10 µl) was then added to the cells for 4 h, and the optical density was detected at 490 nm using a Tecan microplate reader (Infinite F50; Tecan Group, Ltd.).

### 2.4 Western blot analysis

Human tissues and cell samples were prepared using RIPA lysis buffer. Forty nanograms of protein sample was loaded onto an SDS–PAGE gel and transferred to a nitrocellulose membrane. The membrane was blocked with 5% nonfat milk and incubated with primary antibodies overnight at 4°C: rabbit anti-URB2 (1:1000, HPA008902, Merck); rabbit anti-PCNA (ab92552; 1:1000; Abcam); and rabbit anti-β-actin (ab115777; 1:5000; Abcam). The membranes were incubated with the corresponding secondary antibody for 2 h.

### 2.5 Dataset acquisition and processing

To analyze the glioma patient characteristics, the clinical data were obtained from the CGGA database (http://www.cgga.org.cn/about.jsp). The protein expression profiles were obtained from the CPTAC database (https://cptac-data-portal.georgetown.edu/datasets) (Zhang et al., 2016). We considered OS as the primary outcome. Using the R programming language, the URB2 gene expression data and standardized RNA‐seq data were compared. We applied box plots to display the expression difference of discrete variable visualization, and R 4.1.1 (https://www.r-project.org/) was used to perform all the analyses. To investigate the differences in URB2 mRNA expression levels in TCGA glioma patients, the R package “Limma” was applied. Additionally, an adjusted *p*-value (FDR) < 0.05 and |log2-fold change (FC)| ≥1 were considered statistically significant.

### 2.6 Chemotherapy sensitivity analysis

To evaluate NCI-60, we used the CellMiner (https://discover.nci.nih.gov/cellminer/) database (Reinhold et al., 2012). We used Pearson correlation analysis to determine whether the expression of URB2 was associated with drug sensitivity in the model.

### 2.7 Gene set enrichment analysis (GSEA)

By using GSEA, we can determine gene sets of hallmarks that significantly differ between the two groups (low and high URB2 expression). We performed GSEA to examine the significance of differences in survival between the two groups. A 1000-fold permutation of gene sets was performed for each analysis to determine significant biological pathways. The pathways were considered significant when the nominal *p* values < 0.05 and |normalized enrichment score (NES)|>1.5.

### 2.8 Single-cell data analysis

We downloaded the raw data of GSE103224 and GSE148842 from the TISCH database, which were derived from two articles on single-cell sequencing of gliomas (Yuan et al., 2018; Zhao et al., 2021). After a series of dimensionality reduction clustering and corresponding cell annotation, we annotated each cell population into specific cells and showed the expression of the gene in each cell type using UMAP and violin plots, respectively.

### 2.9 Independent prognostic factor evaluation and nomogram construction

Cox regression analysis was applied in our model to examine the association between OS and variables and its independent prognostic value. We also confirmed the related gene URB2 expression. To visualize the relationship between survival rates and individual predictors, a nomogram-based model was constructed by the R “rms” package. Through the “survival ROC” package in R, we evaluated the prognostic ability by AUC and ROC analysis.

### 2.11 Immune correlation analysis

The correlation between URB2 expression and tumor mutational burden (TMB) was calculated by the Pearson correlation coefficient. The same calculation procedure was used for microsatellite instability (MSI) and tumor neoantigen burden (TNB). By analyzing the TIMER (https://cistrome.shinyapps.io/timer/) database, the relationship between URB2 expression and CD8 T-cells, B cells, macrophages, CD4 T-cells, dendritic cells, and neutrophils was determined. To explore the composition of the TME, we assessed the existence of infiltrating immune cells in glioma and calculated the ESTIMATEScore, which was estimated by expression data. To investigate the association between immunity and glioma progression, we profiled the expression of immune cells and immune checkpoints in glioma patients in TCGA datasets.

### 2.12 Statistical analysis

Analysis of all statistical data and figures was performed using R 4.1.1. (https://www.r‐project.org/). The Pearson correlation method was used to analyze the correlation between two genes. The Wilcoxon signed rank test and logistic regression were applied to estimate the relationship between URB2 and clinicopathological characteristics. The log-rank test and Kaplan‒Meier (KM) curve were applied to confirm the risk score (RS) and survival predictive ability of URB2. In this study, statistical significance was determined by *p* > 0.05.

## 3 Results

### 3.1 URB2 expression and its relationship to overall survival in glioma, as validated by other datasets

**FIGURE 1:**
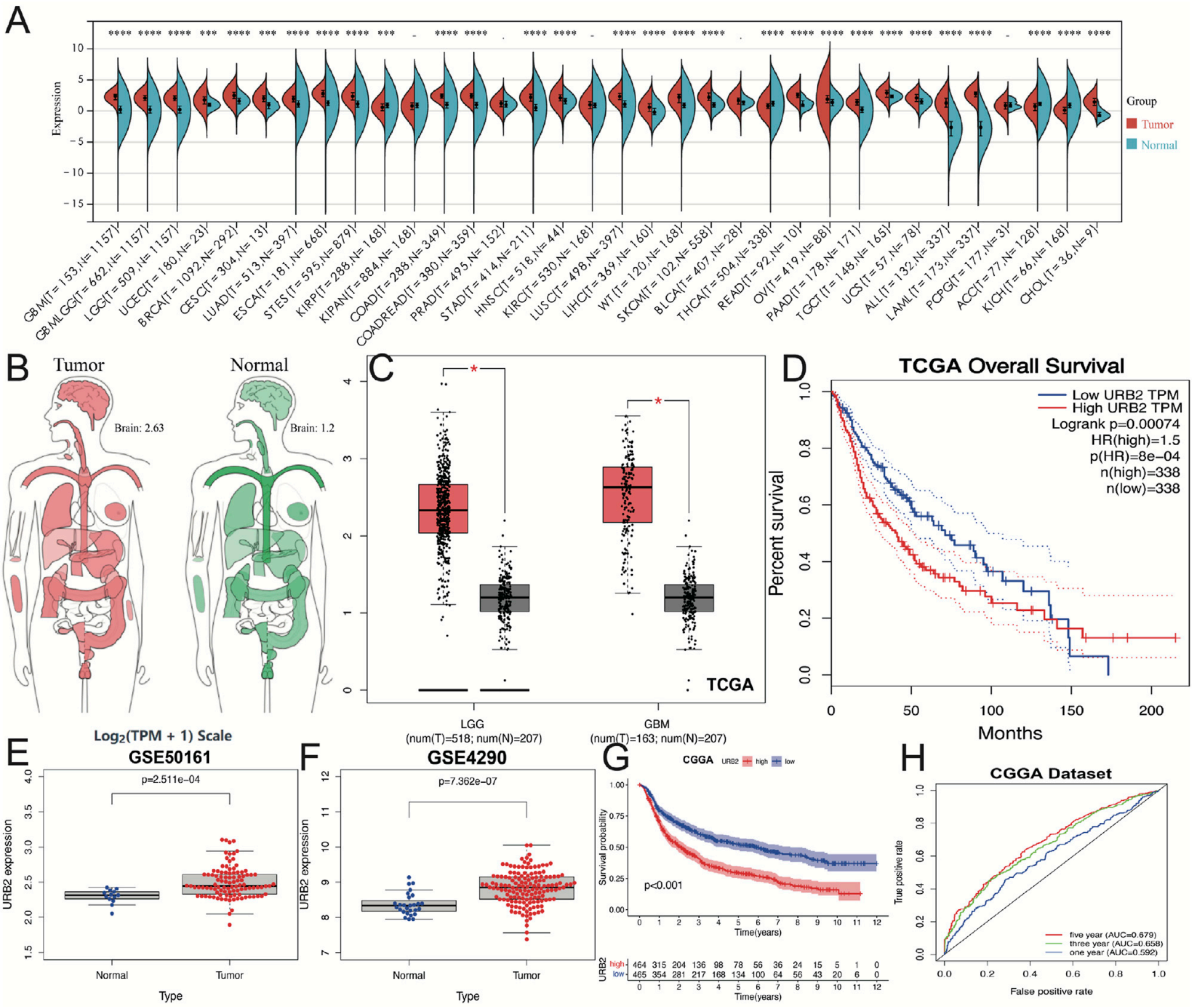
URB2 is overexpressed in glioma. Differences in the expression of URB2 in various cancers and normal tissues in the TCGA database **(A)**. Differential expression of URB2 between normal and tumor tissue in brain **(B)**. Differential expression of URB2 between LGG/GBM and normal tissue **(C)**. Overall survival of glioma patients in high and low URB2 expression groups from the TCGA database **(D)**. Differential expression of URB2 between normal and tumor tissue in GEO database **(E–F)**. KM survival curve of URB2 in CGGA dataset **(G)**. ROC curves associated with 1-, 3-, and 5-year AUC values of URB2 in CGGA dataset **(H)**. *p < 0.05; ***p < 0.001; ****p < 0.0001.

Figure 1A shows the expression levels of URB2 mRNA across all types of cancer in the TCGA study, which illustrates the high expression of URB2 either in GBM or LGG compared with normal tissue. By analyzing the GEPIA (http://gepia.cancer-pku.cn/) database, we constructed human tissue-enriched mRNA expression maps for URB2 in a more intuitive manner (Figure 1B). URB2 expression was markedly higher in both GBM and LGG than in normal tissue (*p* < 0.05; Figure 1C). Based on the median expression level, URB2 expression was divided into low and high groups. Then, the KM curves indicated that the high URB2 expression group had a worse OS than the low URB2 expression group in the TCGA database (*p*-value < 0.01; Figure 1D). Similar results were found in the GEO datasets GSE50161 and GSE4290 (both *p* < 0.01, Figures 1E, F). Moreover, a higher expression of URB2 was associated with worse OS, as validated in the CGGA database (Figure 1G). To further assess the diagnostic ability of URB2, we conducted a receiver operating characteristic (ROC) curve analysis, and the area under the curve (AUC) was 0.592 (1-year), 0.658 (3-year), and 0.6790 (5-year), respectively, indicating a low efficacy in diagnosing glioma based on the expression of URB2 (Figure 1H).

### 3.2 Protein expression of URB2 in glioblastoma multiforme in the CPTAP database

**FIGURE 2:**
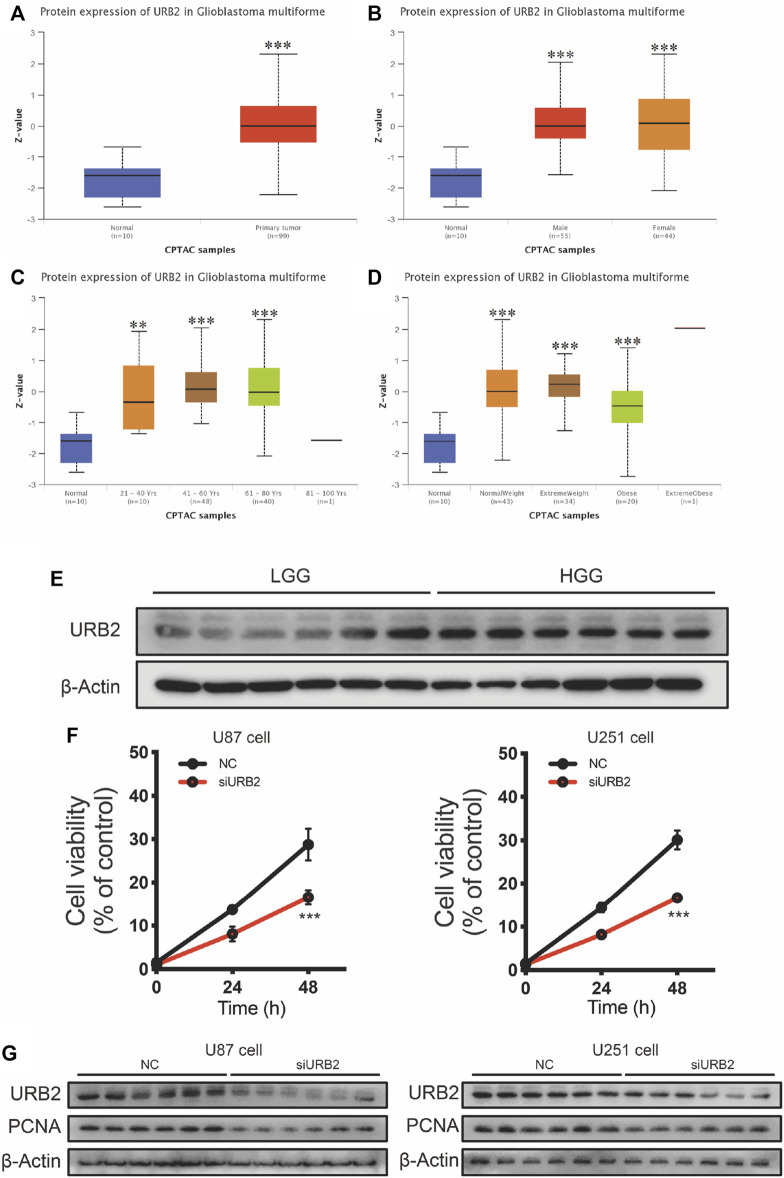
Protein expression of URB2 in Glioblastoma multiforme by CPTAP analysis and validated by Western blot. Differential expression of URB2 protein between normal tissue and primary glioblastoma multiforme **(A)**. Differential expression of URB2 protein among primary glioblastoma multiforme in different genders (male and female) and normal tissue **(B)**. Differential expression of URB2 protein among primary glioblastoma multiforme at different ages (21–40 years; 41–60 years; 61–80 years; 81–100 years) and normal tissues **(C)**. Differential expression of URB2 protein among primary glioblastoma multiforme at different weight (normal weight, extreme weight, obese, and extreme obese) **(D)**. URB2 protein expression levels in GBM and LGG **(E)**. The cell proliferation after downregulation of URB2 in U87 and U251 cells **(F)**. The expression of PCNA after downregulation of URB2 in U87 and U251 cells **(G)**. **p < 0.01; ****p < 0.001.

To demonstrate the difference in the protein expression level of URB2 between normal brain tissues and glioma, we further validated the CPTAC database (Figure 2). In CPTAP samples, URB2 protein expression was much higher in gliomas (Figure 2A), and similar results were found in glioma patients of different sexes (Figure 2B), ages (Figure 2C) and weights (Figure 2D).

Then, we also tested the expression of URB2 in low-grade glioma (LGG) and high-grade glioma (HGG). According to the results of Western blot, it can be observed that the expression of URB2 was significantly higher in HGG than LGG (Figure 2E). To further explore the role of URB2 in the progression of glioma, we downregulated the expression of URB2 in U87 and U251 cells. Of note, the cell proliferation was markedly inhibited after downregulation of URB2 in both cells (Figure 2F). Consistent with the results of cell viability, downregulation of URB2 in both cells can inhibited the expression of PCNA, which also indicated the inhibited cell proliferation (Figure 2G).

### 3.3 Clinicopathological variables and overall survival are correlated with URB2 expression

**FIGURE 3:**
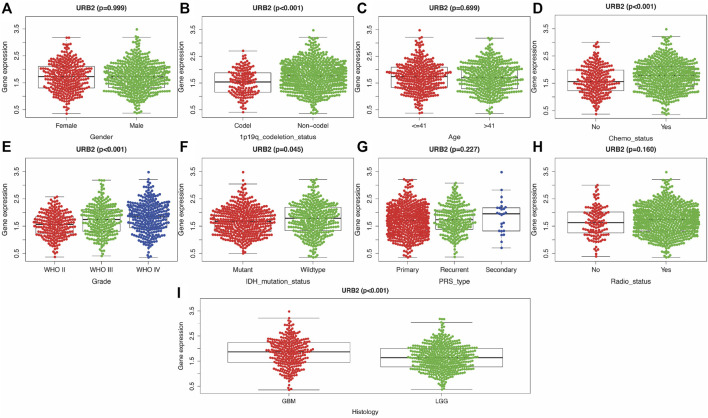
Relationship between clinicopathologic characteristics and overall survival of URB2 **(A–I)**. Correlation of URB2 expression with Gender **(A)**, 1p/19q codeletion status **(B)**, Age **(C)**, Chemo status **(D)**, Grade **(E)**, IDH mutation status **(F)**, PRS type **(G)**, Radio status **(H)**, and Histology **(I)**.

Independent-samples t tests were used to evaluate the clinical meaning of URB2 expression. We revealed that the URB2 expression level was significantly correlated with 1p/19q codeletion status (Figure 3B), Chemo status (Figure 3D), grade (Figure 3E), IDH mutation status (Figure 3F), and histology (Figure 3I) while there was no correlation in gender (Figure 3A), age (Figure 3C), RAS_type (Figure 3G), and Radio status (Figure 3H).

**FIGURE 4:**
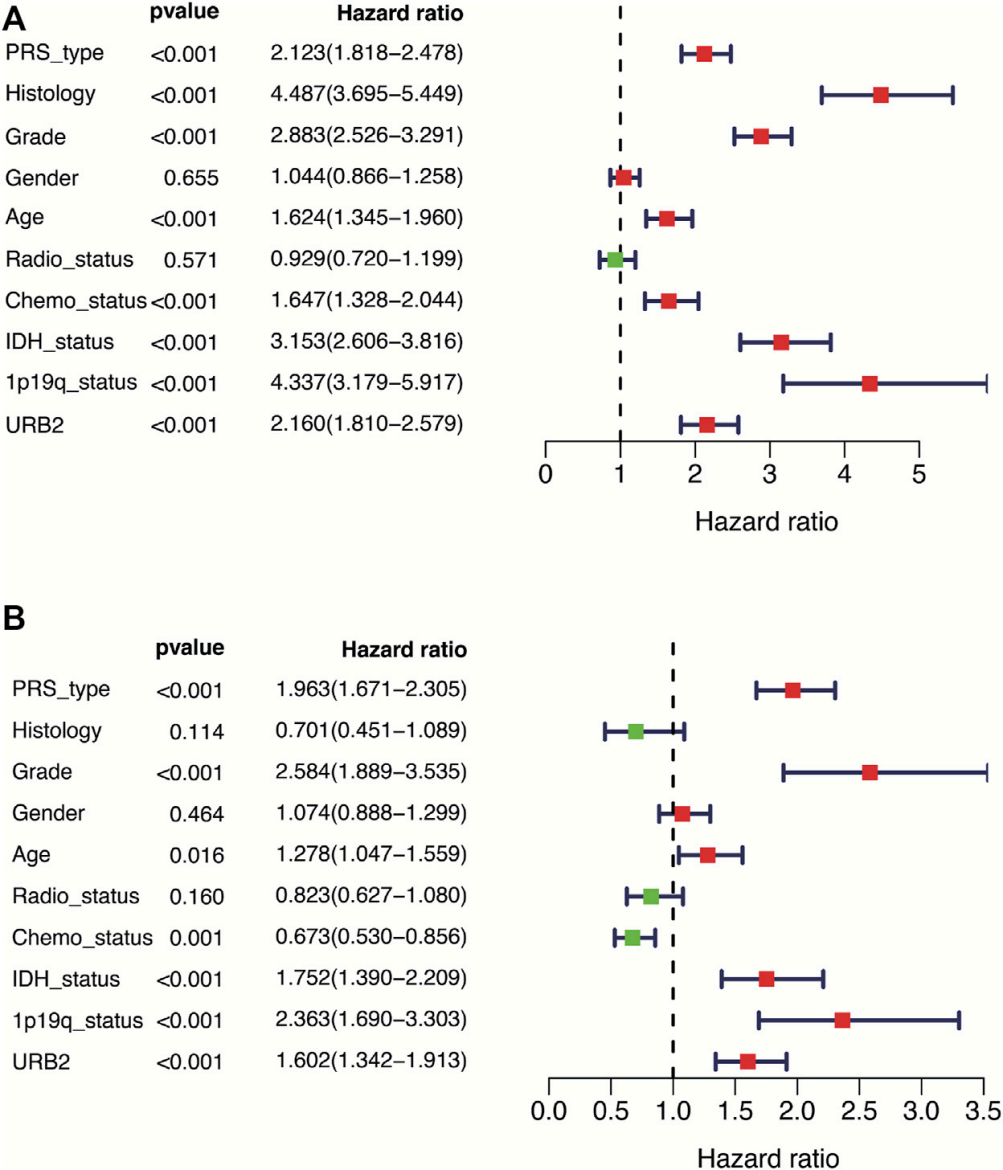
Forest plot showing univariate and multivariate cox regression analyses. Forest plot showing univariate and multivariate cox regression analyses of URB2 mRNA levels and clinicopathological variables predictive of overall survival **(A, B)**.

Cox regression analysis revealed that the URB2 expression level can be used as an independent prognostic risk factor related to OS (Supplementary Table S1). Univariate Cox analysis indicated that PRS type, histology, 1p/19q status, age, grade, IDH mutation, Chemo status, and URB2 expression were significantly related to OS in glioma patients (Figure 4A). In addition, multivariate Cox regression analysis revealed a large negative correlation between URB2 expression and OS (HR = 1.602; *p* < 0.001). Some parameters associated with worse OS included Chemo status, PRS type, IDH mutation, grade, 1p/19q status, and age (Figure 4B). The analyses suggest that URB2 expression can be used as an independent prognostic factor for OS.

### 3.4 Establishment of nomogram for prognosis prediction of glioma

**FIGURE 5:**
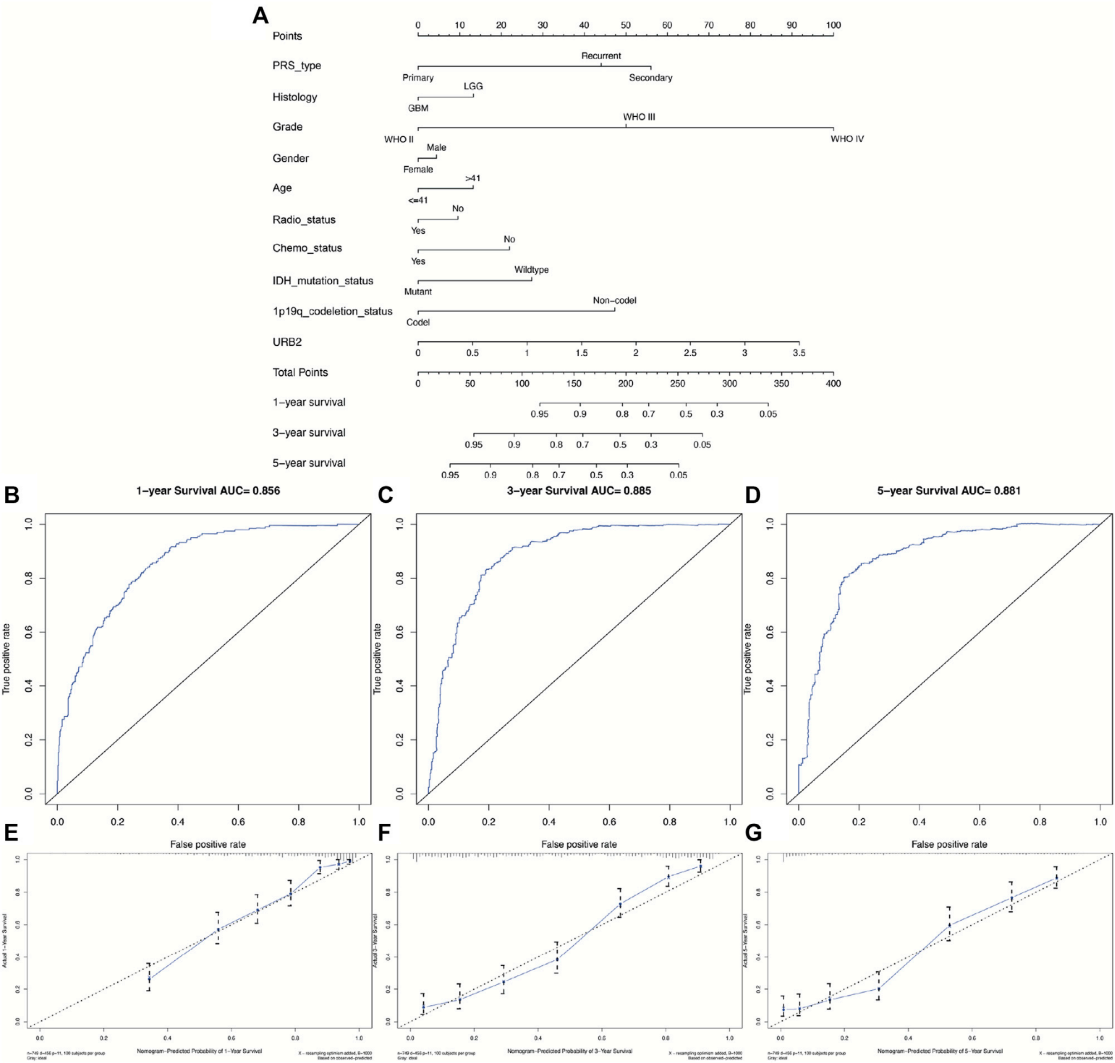
Evaluation of URB2 expression as a prognostic indicator for glioma. The nomogram uses clinical parameters and expression of URB2 to predict overall survival for glioma patients **(A)**. Analyses of the ROC curves for the OS of URB2 expression in the CGGA cohort over a 1-year, 3-year, and 5-year period **(B–D)**. An analysis of the nomogram for the prediction of survival over time **(E–G)**.

By constructing a nomogram that incorporates both clinicopathological variables and URB2 expression, we introduced a quantitative method to predict prognostic risk (Figure 5A). ROC analysis was also performed to determine the prognostic value of URB2 expression in gliomas, in which the AUC of URB2 expression was 0.856 (1-year; Figure 5B), 0.885 (3-year; Figure 5C), and 0.881 (5-year; Figure 5D), and the C-index was 0.8009. As shown in Figure 5E–G, the consistency between actual and ideal values is verified. These findings suggest that URB2 in combination with other parameters can be regarded as a predictor to predict the OS of glioma patients, which means that our nomogram is able to predict survival with a medium level of accuracy.

### 3.5 Identification of URB2‐related signaling pathways

**FIGURE 6:**
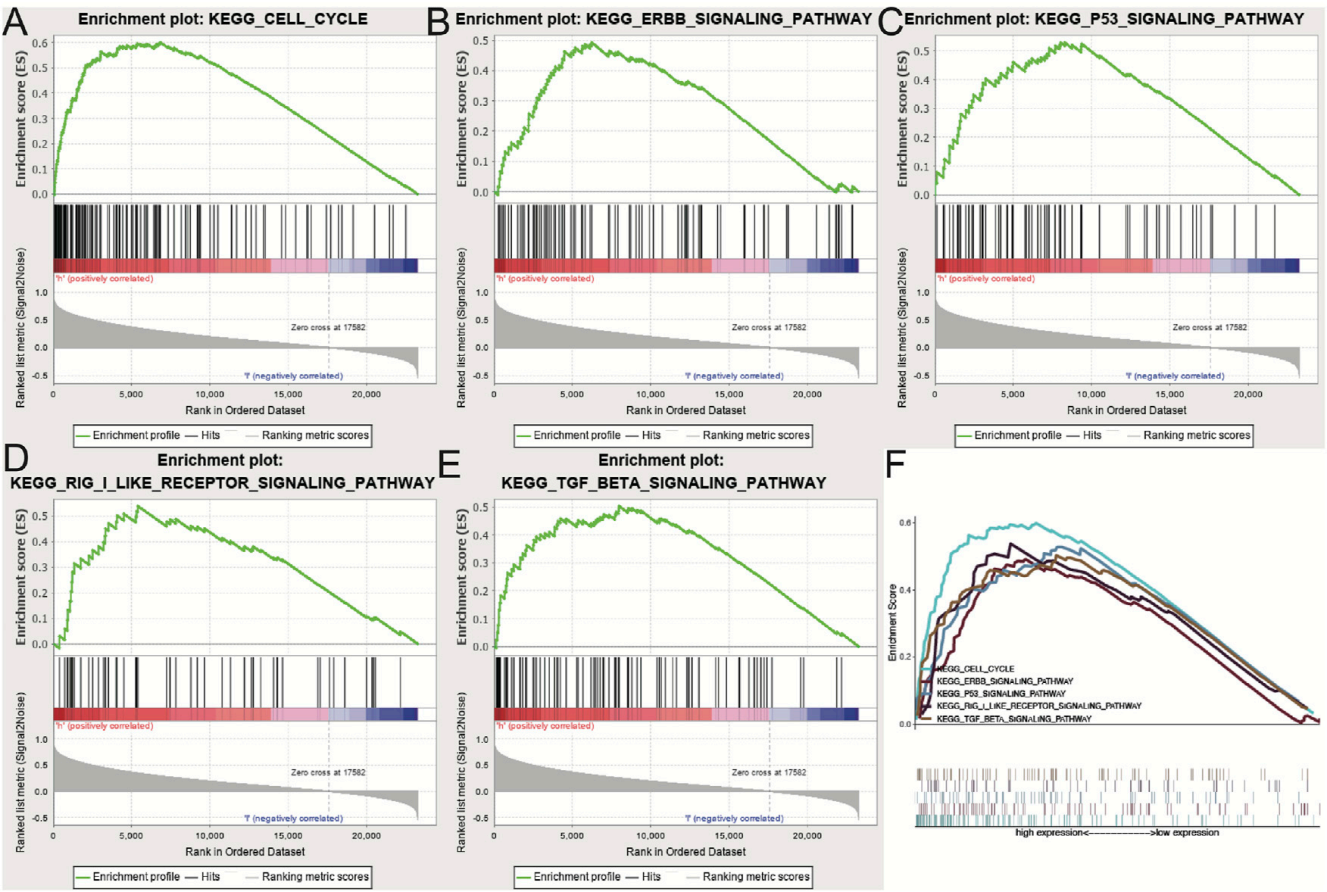
Enrichment of pathways and genes identified by GSEA **(A–E)**. The CELL cycle **(A)**, ERBB signaling pathway **(B)**, P53 signaling pathway **(C)**, RIG I like receptor signaling pathway **(D)** and TGF beta signaling pathway **(E)** are differentially enriched in URB2‐related glioma. **(F)** On the basis of their normalized enrichment score (NES), the five signaling pathways most highly enriched are displayed.

A GSEA was conducted on tissues with varying URB2 expression levels to identify pathways potentially related to URB2. Based on NES and Nom *p*-val <0.05, the pathways that were most substantially enriched were identified. High expression of URB2 was correlated with several signaling pathways, including the cell cycle, TGF beta signaling pathway, ERBB signaling pathway, RIG I-like receptor signaling pathway, and P53 signaling pathway (Figure 6) (NES, normalized enrichment score; and Nom *P*‐val, normalized *p*‐value).

### 3.6 Associations between URB2 and TMB, TNB, MSI, and PPI

**FIGURE 7:**
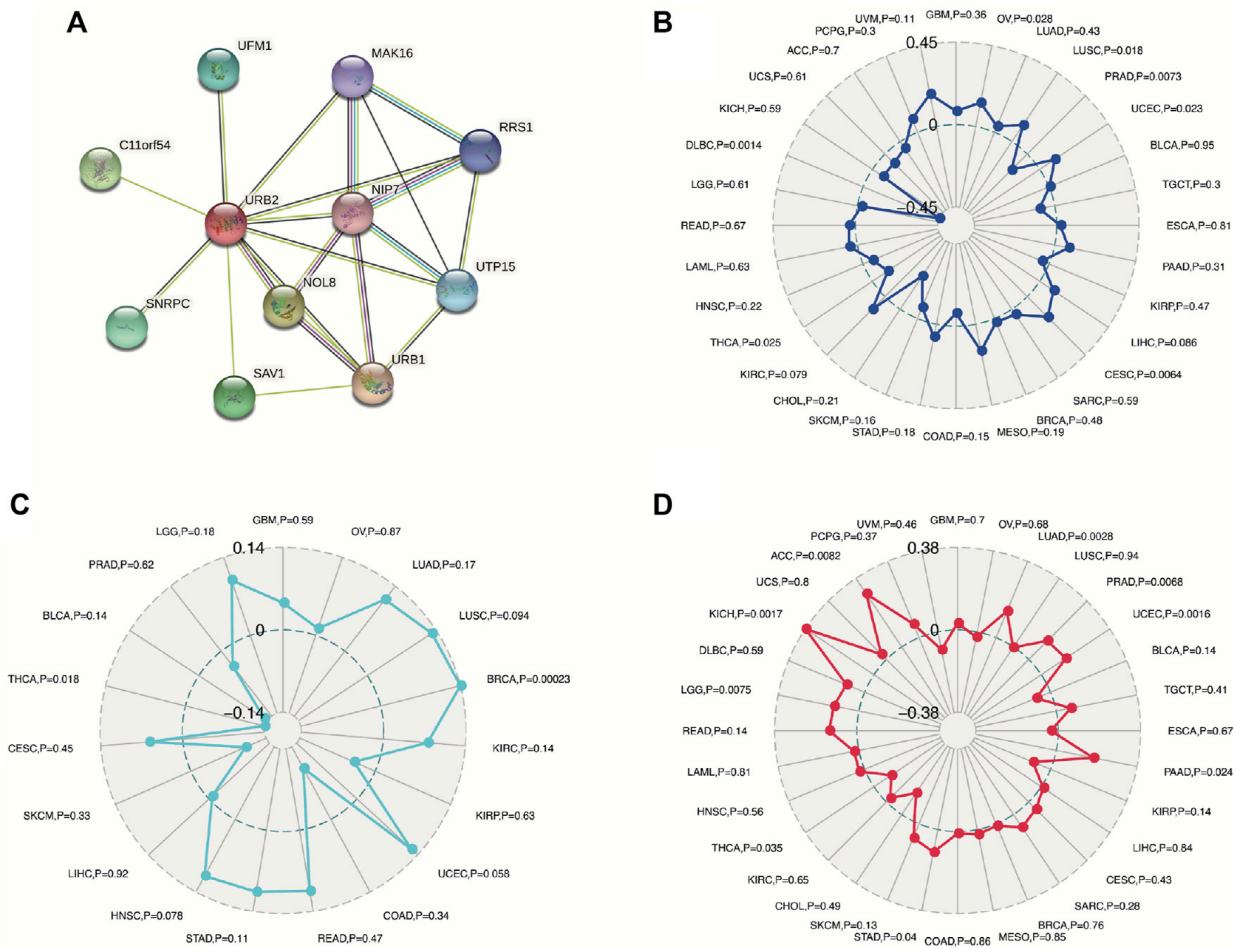
Associations between URB2 and PPI **(A)**, MSI **(B)**, TNB **(C)**, and TMB **(D)** in TCGA dataset.

The protein‒protein interaction (PPI) network indicated that ten different genes (UFM1, C11orf54, SNRPC, SAV1, NOL8, URB1, NIP7, UTP15, RRS1, MAK16) were significantly related to URB2 (Figure 7A). We also revealed that URB2 was not related to MSI (GBM, *p* = 0.36; LGG, *p* = 0.61), TNB (GBM, *p* = 0.59; LGG, *p* = 0.18), or TMB in GBM (*p* = 0.7) (Figures 7B–D), while URB2 was related to TMB in LGG (*p* = 0.0075) (Figure 7D). Thus, in gliomas, TMB may play an important role in URB2 function.

### 3.7 Relationships among URB2 and immune infiltrations, the tumor microenvironment, and immune checkpoint molecules

**FIGURE 8:**
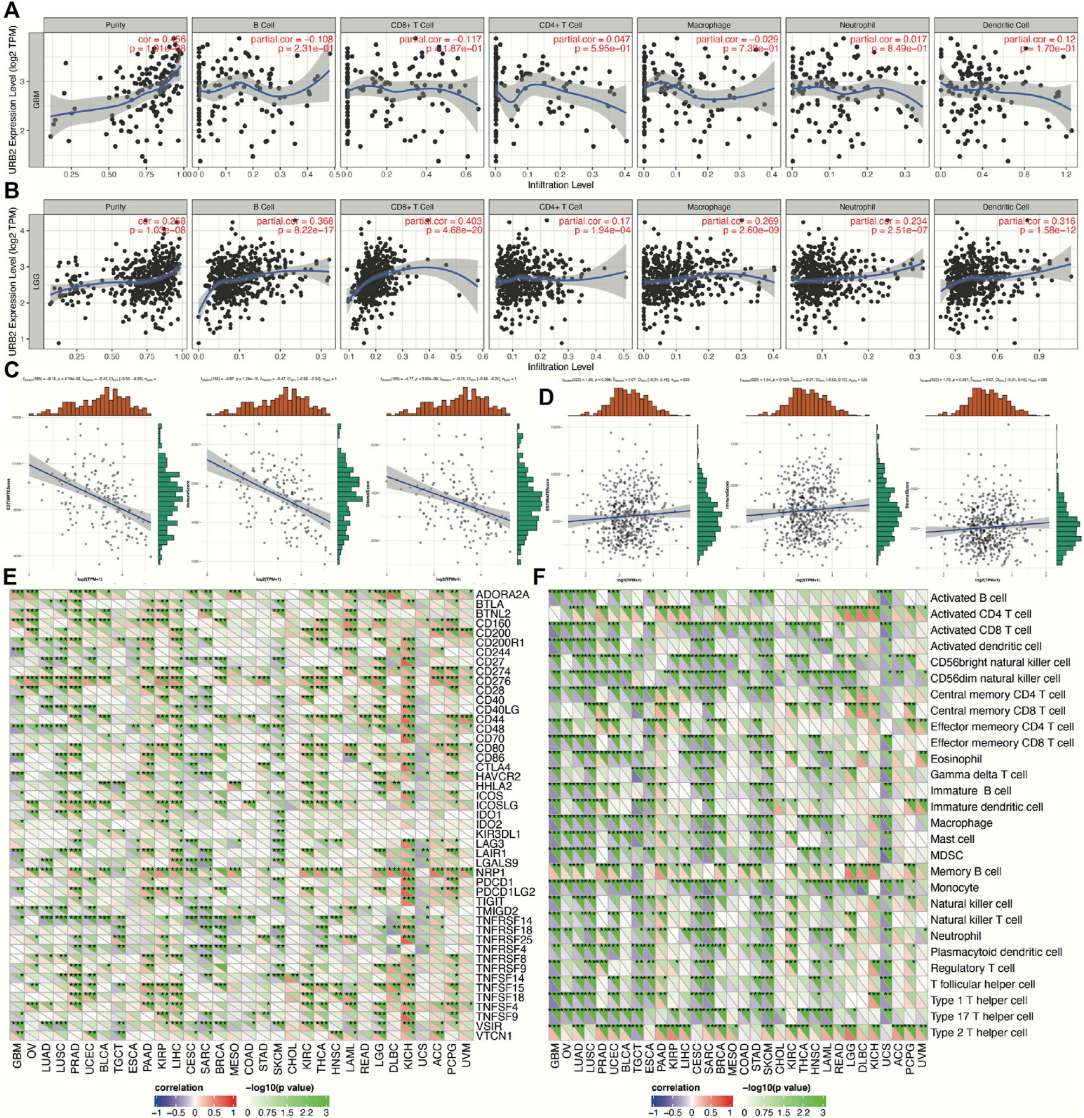
Immune relevance of URB2 in glioma patients. Associations between URB2 and immune infiltrations in GBM **(A)** and LGG **(B)**, tumor microenvironment in GBM **(C)** and LGG **(D)**. Expression of URB2‐related immune checkpoint genes in different tumors **(E)**. Expression of URB2‐related immune cell pathway marker genes in different tumors **(F)**.

We examined the possibility of a relationship between URB2 and the infiltration of six immune cell types using correlation coefficients over 0.3 and *p* values under 0.001. We found that URB2 expression is correlated with none of the six immune cell types in GBM (Figure 8A), while significantly correlated with B cells, CD8^+^ T-cells, and Dendritic cells in LGG (Figure 8B). According to our criteria, URB2 and the immunosuppressive microenvironment of GBM were significantly correlated (Figure 8C), while no correlation was found in LGG (Figure 8D). According to our results, URB2 is significantly correlated with several immune checkpoint molecules in GBM, such as ADORA2A, BTNL2, CD160, CD200R1, and CD244, while the correlated immune checkpoint molecules in LGG include ADORA2A, BTLA, CD160, CD200R1, and CD27 (Figure 8E). GBM also exhibited a significant association with URB2 and several immune cells, such as activated CD8 T-cells, activated dendritic cells, and activated B cells, while it activated CD56dim natural killer cells, central memory CD4 T-cells, and CD4 T-cells in LGG (Figure 8F).

### 3.8 Single-cell data analysis

**FIGURE 9:**
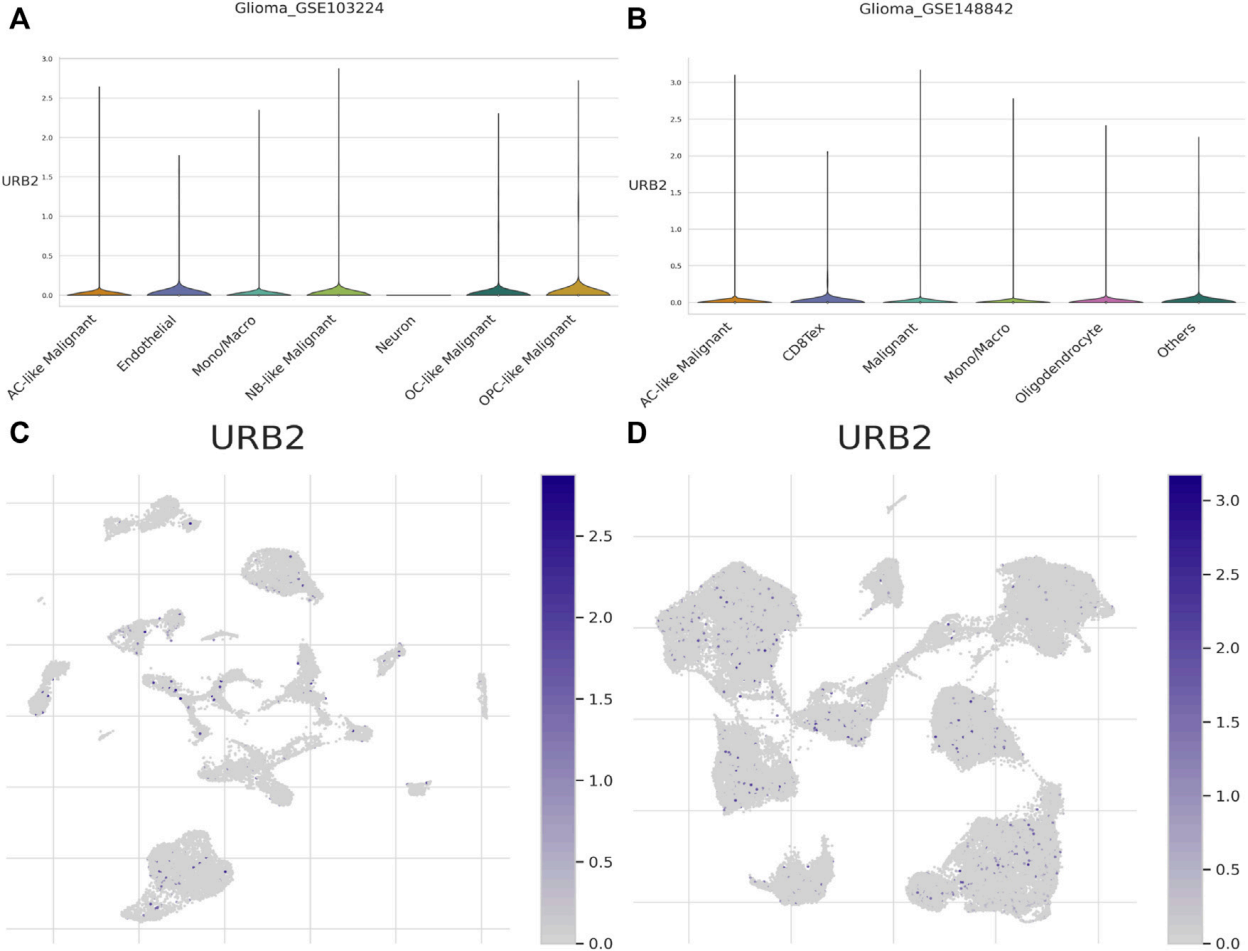
UMAP plots and violin plots. Violin plots of URB2 expression in various types of annotated cells in the GSE103224 and GSE148842 datasets are shown in **(A, B)**, respectively. UMAP plots of URB2 expression in various types of annotated cells in the GSE103224 and GSE148842 datasets are shown in **(C, D)** respectively.

We downloaded the raw data of the GSE103224 and GSE148842 datasets from the TISCH database. After a series of downscaling clustering and corresponding cell annotation, a total of eight cell classes were annotated in the GSE103224 and GSE148842 datasets, which are shown in Supplementary Figures S1A, B. UMAP plots and violin plots of URB2 expression in various types of annotated cells in the GSE103224 and GSE148842 datasets are shown in Figure 9. As is shown in figures, URB2 was expressed in all types of annotated cells, including immune cells, which partially supports the close association of URB2 with immunity in glioma.

### 3.9 Drug sensitivity analysis

**FIGURE 10:**
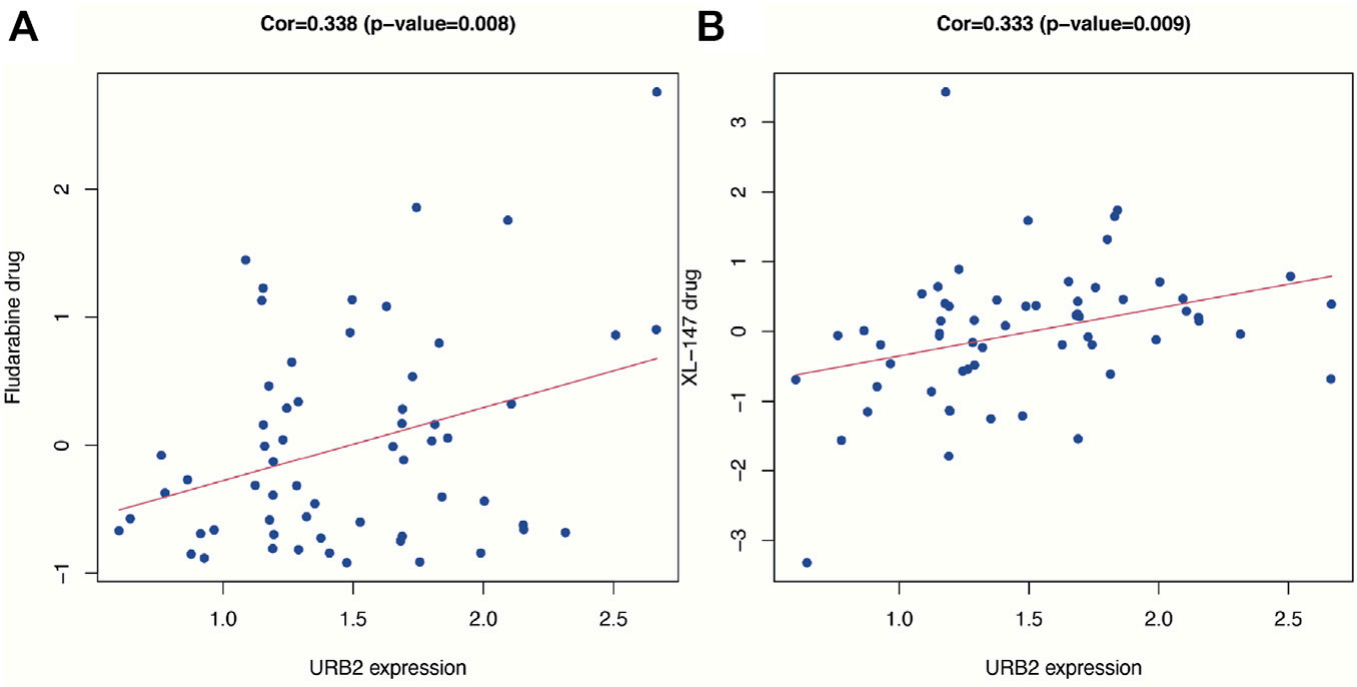
Drug response analysis. The correlation between drug sensitivity (Fludarabine and XL-147) and URB2 in Cellminer database. The scatter plots are ranked by *p*-value.

Figure 10 shows scatter plots demonstrating that drug sensitivity was significantly correlated with URB2 expression (*p* < 0.05). Notably, URB2 has a positive correlation with the sensitivity of fludarabine (correlation coefficient = 0.338, *p* < 0.01, Figure 10A) and XL-147 (correlation coefficient = 0.333, *p* < 0.01, Figure 10B).

## 4 Discussion

As the most frequent primary malignant brain tumor (GBD, 2016 Brain and Other CNS Cancer Collaborators, 2019), glioma claims a large number of lives every year worldwide. While GBM is one of the rarest types of glioma, its poor prognosis still makes it a critically important topic for public health concern (Iacob and Dinca, 2009). In this context, new prognostic targets must be investigated for the prediction of OS and treatment in glioma patients. URb2 is essential for the biosynthesis of 60 S ribosomal subunits. Impairment of URB2 disrupts ribosomal subunits and rRNAs. However, the prognostic role of URB2 and the specific roles of URB2 in tumorigenesis and progression in glioma have not been reported. Therefore, URB2 was evaluated in glioma in terms of prognostic and immunological values in the present study.

In our research, we demonstrated that the expression of URB2 is higher in glioma than adjacent normal tissue, an indication that OS may be poor. This performance has also been verified in the GEO dataset, CGGA dataset, and Western blot (WB) analysis. The protein expression of URB2 in GBM also showed the same result in the CPTAP database. In the CGGA database, low expression of URB2 has a strong correlation with better pathological stage, histological grade, and longer OS in glioma patients. Cox regression analysis revealed that URB2 may be a predictor for prognosis in glioma patients. URB2 expression in patients with gliomas was incorporated with nine clinicopathological variables to generate a risk score, including IDH mutation status, grade, sex, histology, age, radio status, Chemo status, PRS type, and 1p/19q codeletion status. The nomogram also performed well in predicting one-, three-, and 5-year mortality, with AUCs of 0.856, 0.885, and 0.881, respectively. We further performed GSEA between tissues with different URB2 expression levels to explore the role of URB2 in glioma pathogenesis. We found that several key signaling pathways, including the KEGG cell cycle, ERBB signaling pathway, TGF beta signaling pathway, RIG I-like receptor signaling pathway, and p53 signaling pathway, were correlated with URB2 expression. Moreover, we revealed that URB2 expression was strongly associated with the tumor immune microenvironment, immune cell infiltration, immune checkpoint molecules, and immune cells. Using CellMiner, we further found two drugs (fludarabine and XL-147) correlated with URB2, which means that inhibitors of these two drugs can be potential treatment drugs for immune therapy in glioma.

Nomograms are often used in various cancer types to intuitively predict prognosis (Xu et al., 2021a; He et al., 2022). Previous literature has reported that age, chemotherapy status, histopathology, radiotherapy status, IDH, tumor recurrence, and 1p/19q were common prognostic markers in gliomas (Qu et al., 2021; Huang et al., 2022). Our study constructed a nomogram for predicting the OS of glioma patients according to the CGGA dataset based on ten independent prognostic factors, including 1p/19q codeletion status, PRS type, Radio status, Histology, Chemo status, Gender, Age, IDH mutation status, Grade, and URB2. The established nomogram performed moderately with respect to the C-index, ROC curves, and calibration plots with regard to predicting OS for gliomas. Similarly, previous studies have been conducted to predict patient survival by constructing prognostic models for glioma with satisfactory results. By constructing a prognostic model such as a nomogram can more accurately predict the prognostic value of patients with glioma (Qu et al., 2020). Overall, we were successful in building an accurate nomogram plot of glioma patient prognosis.

Then, we determined five URB2-related signaling pathways by means of GSEA, including the CELL cycle, RIG I-like receptor signaling pathway, ERBB signaling pathway, P53 signaling pathway, and TGF beta signaling pathway. As reported, ERBB receptor tyrosine kinases play a key role in both normal physiology and cancer. Many epithelial tumors contain mutations of ERBB2, and clinical studies indicate that they are correlated with tumor progression (Hynes and MacDonald, 2009; Xu et al., 2022). When cells are exposed to different stress signals, their p53 signaling pathway is activated, activating several transcriptional programs, including cell cycle arrest, senescence, DNA repair, and apoptosis, leading to tumor growth inhibition (Marei et al., 2021). There are a large number of previous studies on TGF beta signaling pathway. Studies have shown that the TGF-beta signaling pathway has different roles in the different stages of human cancer progression (Manni and Min, 2020; Baba et al., 2022). TGF-beta acts as a cancer suppressor in the initial stage of tumorigenesis (de Caestecker et al., 2000; Zhang et al., 2017; Chandra Jena et al., 2021). Nevertheless, TGF-β acts as a proto-oncogene in the later stage of tumor to promote tumor development (Katz et al., 2013; Huynh et al., 2019). Currently, dysregulation of the TGF-β signaling pathway can be detected in many cancers, such as colon cancer and breast cancer (Sheen et al., 2013; Villalba et al., 2017). In summary, our results reveal potential signaling pathways and biological functions correlated with URB2, which are instructive for further functional studies of URB2.

With regard to the relationship between immunity and URB2, we demonstrate that the expression of URB2 is significantly associated with immune cells, tumor immune microenvironments (TIMs), and immune checkpoint molecules (ICMs). The activation of immune checkpoint blockade appears to be one of the most promising ways to activate therapeutic antitumor immunity (Pardoll, 2012). Additionally, the characterization of the tumor microenvironment (TME) within a patient’s tumor enables us to predict and guide immunotherapeutic responses (Binnewies et al., 2018). Tumor cells can influence the surrounding cells through the TME, which not only facilitates the development of tumor cells, but also evades the surveillance of the immune system and thus affects the therapeutic effect (Quail and Joyce, 2013). In addition to tumor cells, TME also includes non-malignant cells, extracellular matrix, surrounding vascular system, and signaling molecules (Hanahan and Coussens, 2012). TME is characterized by nutrient deprivation, high acidity, hypoxia, and an immunosuppressive microenvironment, through which tumor cells are able to consolidate their advantage and gain a competitive position (Shi et al., 2020). Immunotherapy for tumors, which is the activation of the body’s anti-tumor immunity, including ICIs, T-cell transfer therapy, monoclonal antibodies, cancer vaccines and immune system modulators, has become one of the most promising and advanced anti-cancer strategies (Topalian et al., 2020). Immunotherapy is dependent on the interaction between tumor cells and immune cells in TME. In addition, the development of nanotechnology and nanomaterials also provides powerful tools for immunotherapy of tumors. Some of these biomaterials (e.g., dendrimers) can be used as carriers for immunologically active drug delivery in cancer through implantation, injection, and transdermal delivery, providing a more advanced approach to immunotherapy (Cai et al., 2020; Gao et al., 2021). Local delivery of immunotherapy through these materials can activate the immune response, reduce the drug dose and achieve high efficacy and safety of the treatment. In some latest studies, nano adjuvants have been used to enhance immunotherapy response and boost anti-tumor immunity through synergistic light-mediated immunotherapy (Zhu et al., 2023). Because of its high specificity and long-lasting antitumor effects, light-mediated immunotherapy has been regarded as a promising therapy for cancer treatment (Monaco et al., 2022). As a result, tumor immunotherapy has been seen as a method for controlling and eliminating cancer. It has been shown that cancer immunotherapy, in particular ICI, has yielded very promising clinical results for a wide range of cancer types, which has triggered considerable interest as a new therapeutic approach for glioma (Assi et al., 2018). Rather than directly killing tumors, immunotherapeutic drugs enhance the human immune system, which results in more effective tumor death and longer-lasting cancer remission while causing fewer side effects.

Furthermore, a correlation was also found between the expression of URB2 in six immune-infiltrating cells taken from the TIMER database. Previous studies have revealed that tumor-infiltrating immune cells (TIICs) play a key role in glioma patients (Liu et al., 2017). TIIC is part of the complex microenvironment. More specifically, it plays a critical role in promoting or inhibiting tumor growth (Domingues et al., 2016). In this research, we evaluated immune infiltration based on URB2 expression and demonstrated that URB2 expression positively correlated with B cells, CD8^+^ T-cells, and Dendritic cells in LGG; however, no correlation was found in GBM. Then, we evaluated the StromalScore, ImmuneScore, and ESTIMATEScore to determine whether URB2 expression correlates with the microenvironment around gliomas. The URB2 phenotype may be associated with immune suppression in GBM but not in LGG, as we found immune involvement in GBM but not in LGG. Furthermore, several immune checkpoints that have been implicated in gliomas were evaluated and associated with URB2 using immune checkpoint analysis. Multiple immune checkpoints correlated significantly with URB2 in LGG as well as GBM, suggesting that immune therapy could be targeted at some of these immune checkpoints. Several immune cells associated with URB2 in gliomas were expressed. These findings showed that gliomas are associated with a dysfunctional immune system, given that the microenvironment in which gliomas develop is immunosuppressive. We further found two drugs (fludarabine and XL-147) with a correlation with URB2, which means that inhibitors of these two drugs can be potential treatment drugs for immune therapy in glioma.

In recent years, research on single-cell sequencing and single-cell data analysis has become very popular and has been used in various tumor studies, especially in brain tissue (Wouters et al., 2020; Zhang et al., 2021). In the study of tumors, it can identify the tumor and immune microenvironment, the heterogeneity of the tumor, and the mechanisms associated with the development and evolution of the tumor (van Galen et al., 2019; Zilionis et al., 2019). In breast cancer, for example, scRNA-seq can examine the multi-omic features of individual cells, thus mapping tumor microenvironment (TME) in breast cancer, which also supports precise treatment. In glioma, the spatial, molecular, and functional heterogeneity of tumor-associated immune cells can be investigated to identify immunotherapeutic targets (Abdelfattah et al., 2022). In conclusion, we can better understand the molecular characteristics of glioma by scRNA-seq, which is important for the development of new therapeutic strategies.

In addition, microsatellite instability (MSI) is defined as MMR-impaired DNA mismatch repair (MMI) causing genetic hypermutability. Genetic hypermutability results from impaired DNA mismatch repair (MMR). The presence of MSI indicates that the function of MMR is not normal (Boland and Goel, 2010). MSI is associated with all types of cancers, including brain cancer (Eckert et al., 2007; Latham et al., 2019), even if MSI phenotyping appears to be closely linked with specific clinicopathological features, primarily in colorectal cancer (Boland and Goel, 2010). Screening for gene mutations in MSI and MMR has been seen as important in the treatment of patients with glioma (Leung et al., 1998; Xu et al., 2021b). Thus, we analyzed the correlation between MSI and the expression of URB2 in glioma. Our results showed no association between MSI and URB2 expression in either GBM or LGG, with *p* values of 0.36 and 0.61, respectively. In many cancer types, tumor mutational burden (TMB) can be used as a biomarker (Johnson et al., 2017). Our results indicated that URB2 expression had no correlation with TMB in GBM, with *p* values of 0.7, but had a significant association with TMB in LGG (*p* = 0.0075).

Last, this study has several highlights. In addition to being discovered in the CGGA dataset, URB2 expression has also been verified in the TCGA dataset, GEO database, and Western blot analysis, which makes our results more reliable. Furthermore, we not only identified the correlation between URB2 and immunity through multiple perspectives but also identified immunotherapeutic agents targeting URB2 in glioma. Most importantly, this is the first study of the prognostic role of URB2 and the immunological role of URB2 in tumorigenesis and progression in glioma. This study also has some limitations, such as the lack of clinical information. Aside from tumor biology, several other factors can also affect the prognosis of glioma patients, including the clinical medical data related to their treatment center. Thus, the role of the URB2 gene has not been fully investigated, and few previous articles have discussed this. The specific role of URB2 in glioma has not been fully investigated experimentally. Therefore, there is a strong need for further experimental work to verify the prediction.

## 5 Conclusion

Together, our research indicated that URB2 plays an oncogenic role in gliomas. According to Cox regression analyses, URB2 was considered an independent factor for glioma. GSEA was applied to search for URB2-associated pathways, including the ERBB and P53 signaling pathways. Additionally, the nomogram we performed demonstrated that URB2 may be a valid predictor, whether alone or in combination with other clinical factors. More importantly, a close relationship between immunity and URB2 was found, which is preliminary and underling evidence that the immune response contributes to glioma progression, suggesting novel approaches to immune therapy for glioma. Finally, further *in vitro* and *in vivo* experiments are necessary to verify our results.

## Data Availability

The original contributions presented in the study are included in the article/Supplementary Materials, further inquiries can be directed to the corresponding authors.
