# Supplementary material for: URB2 as an important marker for glioma prognosis and immunotherapy
Source: Front Pharmacol. 2023 Mar 24;14:1113182. doi: 10.3389/fphar.2023.1113182 (PMC10080038; doi:10.3389/fphar.2023.1113182)
Supplement: Supplementary file 1 [file Table1.DOCX]

| **Factors** | **Univariate analysis** | | | | **Multivariate analysis** | | | |
| --- | --- | --- | --- | --- | --- | --- | --- | --- |
|  | **HR** | **HR.95L** | **HR.95H** | **pvalue** | **HR** | **HR.95L** | **HR.95H** | **pvalue** |
| PRS_type | 2.12258209 | 1.81780707 | 2.47845594 | 1.79E-21 | 1.96253985 | 1.67109547 | 2.30481305 | 2.03E-16 |
| Histology | 4.48699145 | 3.69505798 | 5.44865395 | 7.38E-52 | 0.70059587 | 0.45066496 | 1.08913411 | 0.11395372 |
| Grade | 2.88341104 | 2.52641478 | 3.29085283 | 1.44E-55 | 2.58414518 | 1.88891891 | 3.53525304 | 2.89E-09 |
| Gender | 1.04351049 | 0.86553569 | 1.25808117 | 0.65530711 | 1.07382841 | 0.88756527 | 1.29918046 | 0.46365565 |
| Age | 1.62383303 | 1.34516149 | 1.96023579 | 4.49E-07 | 1.27754145 | 1.04709599 | 1.55870348 | 0.01580389 |
| Radio_status | 0.92890947 | 0.71993324 | 1.19854559 | 0.57062349 | 0.82305655 | 0.62739063 | 1.0797453 | 0.15972631 |
| Chemo_status | 1.64738855 | 1.32780684 | 2.04388842 | 5.71E-06 | 0.67347446 | 0.52974275 | 0.85620396 | 0.00124886 |
| IDH_status | 3.15300408 | 2.60553718 | 3.81550292 | 3.84E-32 | 1.75198662 | 1.3895863 | 2.20889996 | 2.11E-06 |
| 1p19q_status | 4.33698759 | 3.179033 | 5.91672415 | 2.08E-20 | 2.36261747 | 1.68999089 | 3.30295349 | 4.92E-07 |
| URB2 | 2.16040807 | 1.80969581 | 2.57908705 | 1.55E-17 | 1.60224644 | 1.34175185 | 1.91331479 | 1.92E-07 |
