# Supplementary figures and images for: URB2 as an important marker for glioma prognosis and immunotherapy
Source: Front Pharmacol. 2023 Mar 24;14:1113182. doi: 10.3389/fphar.2023.1113182 (PMC10080038; doi:10.3389/fphar.2023.1113182)

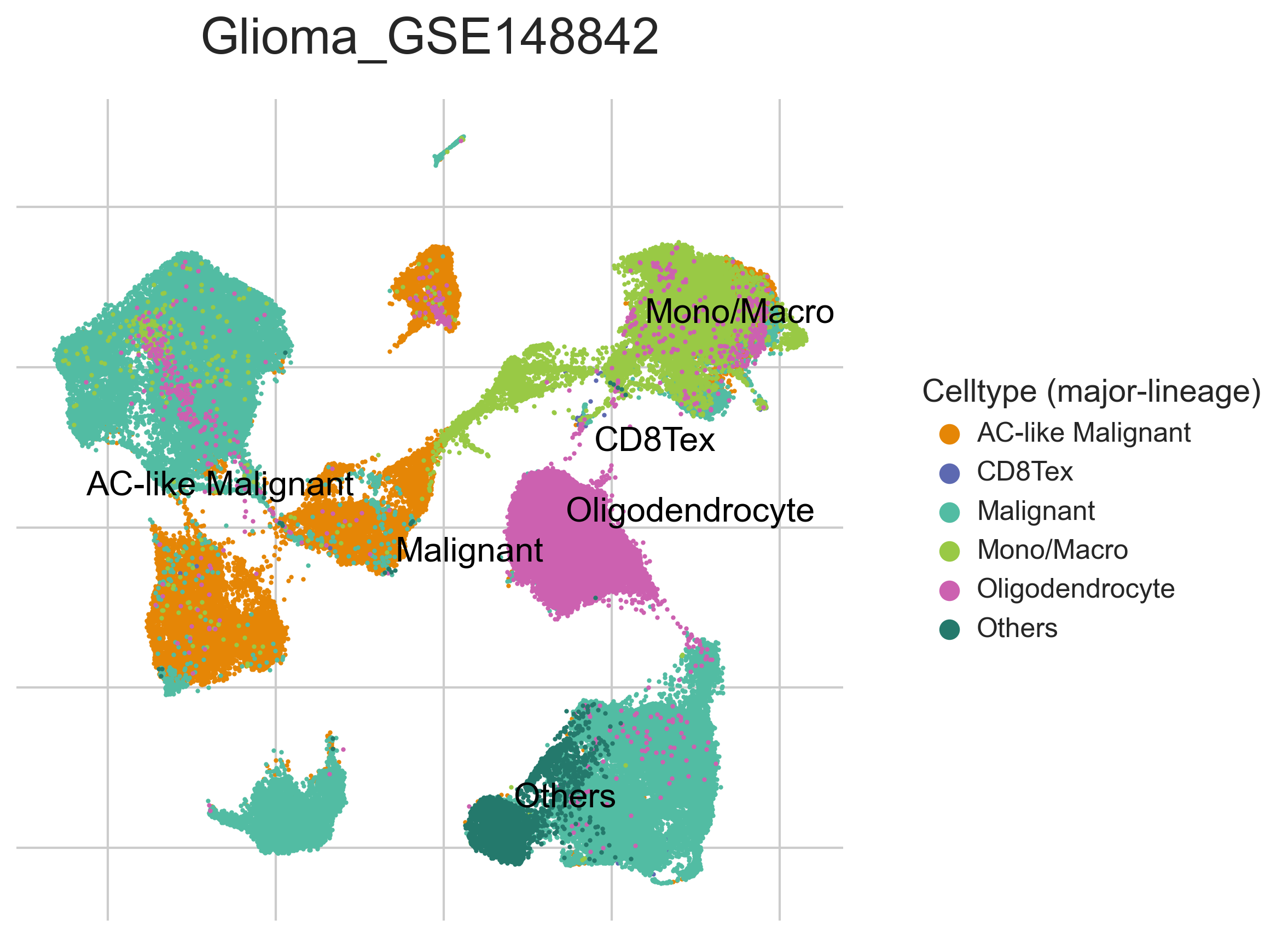

Supplement: Supplementary file 2 [file Image2.png]

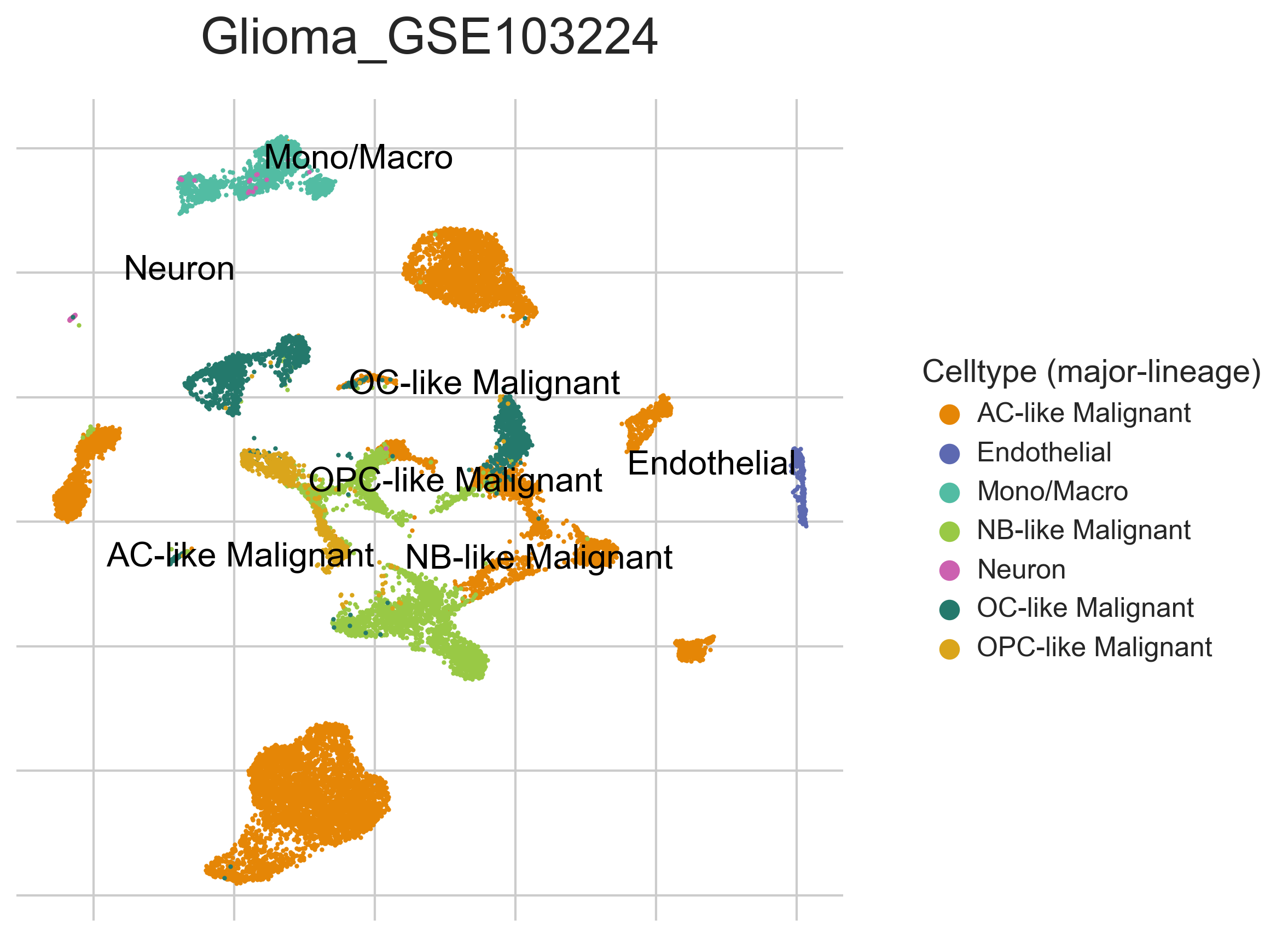

Supplement: Supplementary file 3 [file Image1.png]
